# Congenital Cytomegalovirus Infection: Maternal–Child HLA-C, HLA-E, and HLA-G Affect Clinical Outcome

**DOI:** 10.3389/fimmu.2017.01904

**Published:** 2018-01-05

**Authors:** Roberta Rovito, Frans H. J. Claas, Geert W. Haasnoot, Dave L. Roelen, Aloys C. M. Kroes, Michael Eikmans, Ann C. T. M. Vossen

**Affiliations:** ^1^Department of Medical Microbiology, Leiden University Medical Center, Leiden, Netherlands; ^2^Department of Immunohematology and Blood Transfusion, Leiden University Medical Center, Leiden, Netherlands

**Keywords:** mother–child HLA, congenital CMV infection, immunopathology, allo-reactivity, suboptimal viral clearance, NIMA effect, dried blood spots, biomarkers

## Abstract

Congenital CMV infection (cCMV) is the most common congenital infection causing permanent long-term impairments (LTI). cCMV immunopathogenesis is largely unknown due to the complex interplay between viral, maternal, placental, and child factors. In this study, a large retrospective nationwide cohort of children with cCMV and their mothers was used. HLA-C, HLA-E, and HLA-G were assessed in 96 mother–child pairs in relation to symptoms at birth and LTI at 6 years of age. The mothers were additionally typed for killer cell immunoglobulin-like receptors. The maternal HLA-G 14 bp deletion/deletion polymorphism was associated with a worse outcome, as the immunomodulation effect of higher protein levels may induce less CMV control, with a direct impact on placenta and fetus. The absence of maternal HLA-C belonging to the C2 group was associated with symptoms at birth, as activating signals on decidual NK may override inhibitory signals, contributing to a placental pro-inflammatory environment. Here, the increased HLA-E*0101 and HLA-C mismatches, which were associated with symptoms at birth, may enhance maternal allo-reactivity to fetal Ags, and cause suboptimal viral clearance. Finally, HLA-C non-inherited maternal antigens (NIMAs) were associated with LTI. The tolerance induced in the fetus toward NIMAs may indirectly induce a suboptimal CMV antiviral response throughout childhood. In light of our findings, the potential role of maternal–child HLA in controlling CMV infection and cCMV-related disease, and the clinical value as predictor for long-term outcome certainly deserve further evaluation.

## Introduction

Human CMV is one of the most common causes of congenital viral infection, leading to a significant number of children with hearing loss and neurodevelopmental delay. The overall birth prevalence of congenital CMV infection (cCMV) in industrialized countries lies between 0.6 and 0.7% (1, 2). Among the congenitally infected infants, 12.7% are estimated to have symptoms at birth including petechiae, jaundice, hepatosplenomegaly, thrombocytopenia, chorioretinitis, and microcephaly (1, 2). An estimated 40–58% of these symptomatic children will develop long-term impairments (LTI), such as hearing loss, cognitive, and motor developmental delay (1). Of the 87.3% asymptomatic infants, around 13.5% will also develop LTI (1). Despite the current insights into the clinical outcome of cCMV, the multifactorial process that determines whether a child will be symptomatic at birth or will develop LTI is still poorly understood.

The vertical transmission rate is higher among women without prior CMV infection than among previously exposed women, suggesting a role for maternal immunity in the risk of vertical transmission (2). Vertical transmission takes place *via* placental infection. Once CMV infects the placenta, the extensive local damage and inflammation lead to placental dysfunction, which in turn can impair fetal development (3). The clinical impact of fetal infection is largely determined by vertical transmission in the first 20 weeks of pregnancy, during which the fetal immune system is still developing (4, 5). Importantly, the fetal and neonatal immune system may also play a role in controlling the infection, thereby influencing LTI development (6). A persistent productive infection may lead to late-onset or progression of sensorineural hearing loss, even though a role of immunopathology cannot be entirely excluded (7, 8). Hence, clinical outcome is the result of a multifactorial process that comprises maternal, placental, fetal, and child factors.

Pregnancy is considered a semi-allograft, in that the fetus may have HLA antigens (Ags) that the mother does not have. At the maternal–fetal interface, the majority of trophoblast cells are in direct contact with maternal cells, and several mechanisms are in place to prevent rejection of the fetal semi-allograft. Extravillous trophoblast cells do not express HLA-A, -B, -DR, -DQ, and -DP (9), but they do express HLA-C and the non-classical HLA-E and HLA-G. HLA-C and HLA-E prevent maternal NK cell-mediated cytotoxicity through binding with killer cell immunoglobulin-like receptors (KIRs) expressed on decidual NK cells (dNK). dNK cells are the most abundant leukocyte population in the placenta (9), therefore, they play a fundamental role in pregnancy. KIR genotypes can be distinguished into two haplotypes. The KIR-A haplotype mainly contains inhibitory receptors, such as KIR2DL1, whereas the KIR-B haplotype also contains one or more activating receptors, such as KIR2DS1 (10, 11). NK cytotoxicity is controlled through a balance of both activating and inhibitory receptors on dNK cells (12–15). Furthermore, HLA-G modulates the response of different cellular subsets, including dNK, APC, T cells, and B cells (16, 17). The complex maternal–fetal immune cross-talk at the interface creates a tolerogenic niche for the normal development of the fetus, and it differs from the peripheral immune system of both mother and child (18). Indeed, immune cells can be generated locally with a different function than the one acquired at the periphery. For example, CD8 T cells express significantly lower levels of perforin and granzyme-B, and DC are arrested in a tolerogenic state (18, 19). Each pregnancy is characterized by a unique mother–fetus HLA combination, which may generate different immune effector and regulatory mechanisms (20, 21). Maternal–fetal HLA-C mismatches (mm) induce a shift toward higher levels of effector and regulatory T cells in the decidua (20). In addition, specific combinations of maternal KIRs and fetal HLA-C can induce pregnancy complications (22, 23). Indeed, the HLA-C ligands for KIRs are divided into two groups, the C1 allotype binds inhibitory KIRs whereas the C2 allotype binds both activating and inhibitory KIRs (10, 11). Therefore, while KIR2DL1/HLA-C2 leads to inhibition, KIR2DS1/HLA-C2 leads to activation of dNK (15, 24). The risk of recurrent miscarriage increases in women with KIR-A haplotype when they have fewer C2 genes than the fetus, or when the fetus inherited C2 from the father (18, 25). Indeed, the proper dNK activation is essential to facilitate trophoblast invasion and a successful placentation (26). Furthermore, lower levels of soluble HLA-G (sHLA-G) have been described in the circulation of pregnant women with pre-eclampsia, intrauterine growth retardation, and recurrent spontaneous abortion (27, 28).

With respect to CMV, the role of HLA in the local immune response in the placenta is largely unknown. With regard to HLA-G, higher CMV viral loads in urine of children homozygous for the HLA-G 14 bp deletion were found (29). In addition, higher levels of sHLA-G were shown in serum and amniotic fluid of women with primary CMV infection and in symptomatic neonates (30). The aim of this study was to determine if the combination of maternal and child HLA Ags influences the short- and long-term outcome of cCMV, thus providing new insights into cCMV immune regulation and pathogenesis. Our investigations focused on the role of HLA-C, HLA-E, and HLA-G, which are the only HLA molecules expressed at the placenta.

## Materials and Methods

### Study Population and Clinical Data

A previously described nationwide, retrospective cohort was used in this study (31, 32). The cohort was derived from a total group of 31,484 children, born in 2008 in the Netherlands, which was retrospectively tested for cCMV by PCR of CMV DNA in neonatal dried blood spot (DBS) at 5 years of age. In total, 156 children (0.5%) were diagnosed with cCMV. Clinical data were retrieved from 125 congenitally CMV-infected children and from 263 non-infected children. For this study, buccal swabs from 104 children with cCMV and their mothers were obtained for HLA-typing. Two buccal swabs [FLOQSwabs hDNA Free, 20-mm breaking point in 174.5 mm long dry tube (COPAN ITALIA SPA, Brescia, Italy)] were retrieved from each individual. Children were defined as symptomatic at birth if they had one or more of the following signs or symptoms in the neonatal period: prematurity, being small for gestational age, microcephaly, hepato- or splenomegaly, generalized petechiae or purpura, hypotonia, abnormal laboratory findings (elevated liver transaminases, hyperbilirubinemia, neutropenia, or thrombocytopenia), cerebral ultrasound abnormalities, ophthalmologic abnormalities, or neonatal hearing impairment. LTI was defined as the presence of impairment in one or more domain (hearing, visual, neurological, motor, cognitive, and speech-language). Because in this cohort maternal seroimmunity to CMV before birth was unknown, it was assumed that cCMV infection could have resulted from either maternal primary or secondary infection. This study was approved by the Medical Ethics Committee of the Leiden University Medical Center, and all the parents of the children included have given written informed consent in accordance with the Declaration of Helsinki.

### DNA Extraction from DBS and qPCR of CMV

After a first initial CMV PCR screening performed at the National Institute for Public Health and the Environment (RIVM), a second confirmatory PCR was performed at the Leiden University Medical Center (LUMC) (31). For this purpose, DNA was extracted from DBS by using the QIAamp DNA minikit according to the previously described protocol (33). For each test, one full DBS was punched by using an automated DBS puncher (1296-071, Perkin Elmer-Wallac, Zaventem, Belgium). CMV DNA amplification of a 126-bp fragment from the immediate-early Ag region was performed using an internally controlled quantitative real-time PCR, as described previously (34, 35), on the CFX96 Real-Time PCR Detection System (BioRad, Veenendaal, The Netherlands). The PCR was performed in triplicate, and the CMV viral load was expressed in IU/ml.

### DNA Extraction from Buccal Swabs

DNA was extracted from the buccal swabs by using the QIAamp DNA (blood) mini kit (QIAGEN, Hilden, Germany) according to the manufacturer’s instruction, with an additional PBS pre-incubation of 30 min at room temperature. In addition, the pre-incubation fluid from two swabs was applied to one QIAamp spin column, in order to concentrate the DNA. The DNA was eluted in 150 µl of Tris–EDTA-4 buffer for further analyses.

### HLA and KIR Typing

All mothers and children were DNA typed at low resolution for HLA-C locus (14 alleles) using the Reverse Sequence Specific Oligonucleotides PCR technique. For this purpose, a commercially available assay was applied, LIFECODES C SSO Typing kits (Immucor, Norcross, GA, USA). Data were analyzed using MatchIT software (Immucor-Lifecodes). The HLA-C alleles included in this study were selected based on 5,604 randomly selected healthy Dutch blood donors who were previously genotyped (36). This group is considered to be a proper representation of the gene distribution in the general population (37), and the HLA-C frequencies are depicted in Table S1 in Supplementary Material. HLA-C allotypes were divided in two groups, namely HLA-C1 and HLA-C2, based on the presence of SER77ASN80 (C1) and ASN77LYS80 (C2) at position 80 of the α1 domain, as previously described (38).

All mothers and children were typed for the two most common HLA-E functional alleles, HLA-E*01:01 and HLA-E*01:03 (39). Eight different HLA-E proteins have been identified from 26 coding sequences, but the aforementioned ones, due to their functional differences, are the two most commonly analyzed worldwide (39). Due to a non-synonymous mutation these two functional alleles differ in one aa position in codon 107 of the α heavy chain, HLA-E*01:03 encoded proteins show higher cell surface expression and peptide-binding affinity than HLA-E*01:01proteins (40). Determinations of the HLA-E*01:01/01:03 variants (SNP ID rs1264457) were carried out by using the TaqMan^®^ SNP Genotyping Assays (ThermoFisher Scientific, MA, USA) on a ViiA™ 7 Real-Time PCR System (ThermoFisher Scientific, MA, USA) in a 96 well-plate using a thermocycling profile as follows: 10 min 95°C followed by 40 cycles of 95°C (15 s), 60°C (1 min), and finally after cycling 60°C (30 s) with 1.5 µl of diluted DNA extract and 8.5 µl of reaction mix. Data were analyzed using ViiA™ 7 Software.

All mothers and children were typed for the HLA-G locus by using a TaqMan assay for the 14-bp insertion/deletion polymorphism in exon 8 of the 3′ untranslated region with primers and probes previously described (41). Nine different HLA-G proteins from 28 alleles have been described (42), however, the polymorphism included in this study is one of the most commonly associated with pregnancy (43–47). The TaqMan assay consisted of 1.5 µl diluted DNA extract and 15 µl reaction mixture containing: 7.5 µl TaqMan Universal Master Mix II with UNG (ThermoFisher Scientific, MA, USA), 300 nM of forward and reverse primers, 200 nM of HLAG14FAM (insertion) and HLAGdelCY5 (deletion) probes. The PCR was performed on a Light Cycler^®^96 Detection System (Roche Applied Science, Mannheim, Germany) in a 96 well-plate using a thermocycling profile as follows: 10 min 95°C followed by 50 cycles of 95°C (10 s), 58°C (50 s) and finally after cycling 37°C (30 s). Data were analyzed with Light Cycler^®^96 Analysis Software 1.1.

All mothers were genotyped at low resolution for KIR receptors. The KIR genotype differs based on the absence of activating KIRs (haplotype AA) or on the presence of different numbers of activating KIRs (haplotype AB or BB). In particular, the BB or AB haplotype is defined as the presence of one or more of the following genes: KIR2DL2, KIR2DL5, KIR2DS1, KIR2DS2, KIR2DS3, KIR2DS5, and KIR3DS1. The AA haplotype is defined by the absence of all the above mentioned genes and the presence of the following genes: KIR2DL1, KIR2DL3, KIR2DS4, and KIR3DL1. The KIR genotyping was performed using 11 homemade PCR reactions per individual, according to a previously described protocol (48). In each reaction, 2 µl of diluted DNA extract was amplified in 20 µl of SybrGreen-based PCR mix (IQ Sybr Green Supermix, BioRad, CA, USA) containing 0.3 pmol/μl of primers. The PCR was performed on a Light Cycler^®^96 Detection System (Roche Applied Science, Mannheim, Germany) in a 96 well-plate using a thermocycling profile as follows: 10 min at 95°C followed by 40 cycles of 95°C (15 s) and 62°C (60 s). Data were analyzed with Light Cycler^®^96 Software 1.1.

### Statistical Analysis

Data were analyzed by using the Statistical Package for Social Sciences (SPSS, version 23, Chicago, IL, USA). The chi-square test was used to evaluate the observed and expected genotypes frequency, the number of HLA mm, missing-self (ms), non-inherited maternal antigens (NIMAs), and the combinations between maternal KIR with child HLA-C, in relation to symptoms at birth and LTI. With expected low values, a Fischer’s exact test was used instead. The maternal and fetal HLA allele frequencies were tested for Hardy–Weinberg equilibrium (HWE), stating that in the absence of other influences the genotype frequency in a certain population remains constant from generation to generation (49). In addition, a univariate logistic regression was performed to investigate potential predictors of symptoms at birth and LTI development. A *p*-value <0.05 was considered statistically significant.

## Results

### Study Population and Clinical Data

The clinical data of the congenitally infected children included in this study are depicted in Table 1. A total of 96 mother–child pairs were successfully typed for at least one of the HLA genes included in this study. Eight mother–child pairs could not be typed due to low DNA quality and concentration. Nineteen (20%) children had symptoms at birth, and 11 (58%) of those had LTI. In addition, 16 (21%) asymptomatic children had LTI. Overall, 27 (28%) of infected children developed any LTI.

**Table 1:** Characteristics and clinical outcome of study population.

	Congenital CMV infection		
	Overall	Asympt.^a^	Sympt.^b^
	
	*n* = 96	*n* = 77	*n* = 19
**Gender**			
Male	57	44	13
Female	39	33	6
**Gestational age (weeks)^c^**	39 (28–42)	39 (37–42)	36 (28–41)
**Birth weight (g)****^c^**	3,435 (900–5110)	3,540 (2635–5110)	2,800 (900–4170)
**Long-term impairment**			
Hearing impairment^d^	3	2	1
Visual impairment^e^	2	2	0
Neurological impairment^f^	5	2	3
Motor impairment^g^	13	9	4
Cognitive impairment^h^	7	4	3
Speech/language problem^i^	18	10	8
**One or more impairment****^j^**	27	16	11

*^a^Asymptomatic at birth*.

*^b^Symptomatic at birth: premature (*n* = 11), dysmature (*n* = 2); microcephaly (*n* = 5); neonatal hearing loss (*n* = 1); abnormal cranial ultrasound (*n* = 1)*.

*^c^Values are medians with minimum and maximum*.

*^d^Sensorineural hearing loss*.

*^e^Optic nerve atrophy (*n* = 1), cortical visual impairment (*n* = 1)*.

*^f^Cerebral palsy (*n* = 1), epilepsy (*n* = 1), microcephaly (*n* = 1), ADHD (*n* = 1), autism (*n* = 3)*.

*^g^Motor impairment (fine, gross, or balance) based on test or diagnosis or sensory processing disorder or developmental coordination disorder*.

*^h^Cognitive impairment based on test or diagnosis*.

*^i^Language impairment based on test or diagnosis, speech-impairment, oral motor skill difficulties, or auditory processing disorder*.

*^j^Any long-term impairment, in one or more domains*.

### Maternal and Child HLA-C, HLA-E, and HLA-G Genotypes in Relation to cCMV Clinical Outcome

First, we evaluated whether the maternal genotype plays a role in cCMV outcome. For this purpose, we tested whether the genotype frequencies of the HLA-C groups, C1 and C2, and of the HLA-E and HLA-G alleles from the mothers were in HWE. The HLA-C groups of mothers with children symptomatic at birth were not in HWE (*p* = 0.046) (Table S2 in Supplementary Material). In our cohort, a significantly higher percentage of mothers was homozygous for HLA-C1 in the group of children symptomatic at birth compared to the asymptomatic group, whereas the percentage of mothers homozygous or heterozygous for HLA-C2 was lower (Table 2). The functional difference between HLA-C1 and HLA-C2 suggests a possible association of these alleles with clinical outcome in our cohort (23, 50, 51), with absence of maternal HLA-C2 being a risk factor. Indeed, the odds of having symptomatic neonates was higher when mothers were homozygous for HLA-C1 than when mothers were homozygous for HLA-C2 or heterozygous (OR = 3.950, 95% CI 1.378–11.320, *p* = 0.011). Consequently, only 5.3% of children symptomatic at birth were homozygous for HLA-C2 (Table 2).

**Table 2:** HLA-C, KIR, and congenital CMV infection (cCMV) clinical outcome.

	Symptoms at birth			Long-term impairments (LTI)		
	Symptomatic % (*n* = 19)	Asymptomatic % (*n* = 76)	*p*-Value (Chi)	LTI (≥1)^a^ % (*n* = 26)	No LTI^b^ % (*n* = 69)	*p*-Value (Chi)
**HLA-C genotype**						
*Mother*			0.021*			0.084
C1C2	21.1	52.6		38.5	49.3	
C1C1	63.2	30.3		30.8	39.1	
C2C2	15.8	17.1		30.8	11.6	
*Child*			0.371			0.712
C1C2	52.6	46.1		50	46.4	
C1C1	42.1	35.5		30.8	39.1	
C2C2	5.3	18.4		19.2	14.5	

HLA-C mm^c^	84.2	68.4	0.172	80.8	68.1	0.223
HLA-C1 mm^d^	15.8	11.8	0.701^k^	19.2	10.1	0.300^k^
HLA-C2 mm^e^	31.6	10.5	**0.031**^k^*	19.2	13	0.519^k^

HLA-C ms^f^	78.9	72.4	0.560	96.2	65.2	0.002*
HLA-C1 ms^g^	5.3	13.2	0.454^k^	7.7	13	0.722^k^
HLA-C2 ms^h^	10.5	18.4	0.514^k^	19.2	15.9	0.761^k^

**KIR haplotype mother****^i^**			0.659			0.356
A	36.8	31.5		40	29.9	
B	63.2	68.5		60	70.1	
Maternal KIR A–Child C2^j^	21.1	20.5	1.000^k^	20	20.9	0.925

*^a^Any long-term impairment, in one or more domains of impairments: hearing, visual, neurologic, motor, cognitive, and speech-language*.

*^b^Absence of any long-term impairment*.

*^c^HLA-C mm: HLA-C mismatches (mm)*.

*^d^HLA-C1 mm: HLA-C mm in the C1 group, the mother is homozygous for C2 and the child is heterozygous*.

*^e^HLA-C2 mm: HLA-C mm in the C2 group, the mother is homozygous for C1 and the child is heterozygous*.

*^f^HLA-C ms: HLA-C missing-self*.

*^g^HLA-C1 ms: HLA-C missing-self in the C1 group, the mother is heterozygous and the child is homozygous for HLA-C2*.

*^h^HLA-C2 ms: HLA-C missing-self in the C2 group, the mother is heterozygous and the child is homozygous for HLA-C1*.

*^i^Maternal KIR A haplotype is defined as the absence of activating KIRs (AA) and KIR B haplotype as the presence of different numbers of activating KIRs (AB or BB)*.

*^j^Combination of maternal KIR A haplotype with HLA-C2 positive child (either C1C2 or C2C2); ^i,j^N asymptomatic = 73, N with LTI = 25, N without LTI = 67*.

*^k^Fischer’s exact test used*.

*p < 0.05.

The genotype frequencies of HLA-E and HLA-G alleles from the mothers were in HWE in all groups (Table S2 in Supplementary Material). Despite this, a significantly higher percentage of mothers homozygous for HLA-G deletion (HLA-G del/del) was observed in the group of children that developed LTI compared to those who did not; this percentage was also higher in the symptomatic group compared to the asymptomatic group, though not significant (Table 3). HLA-G del/del is related to higher HLA-G protein levels (52, 53), soluble and possibly membrane-bound; therefore, our findings suggest that the functional difference of the two alleles is associated with clinical outcome, with the maternal homozygous status being a risk factor. Indeed, the odds of developing LTI was higher when mothers were HLA-G del/del (OR 3.542, 95% CI 1.397–8.977, *p* = 0.008). Likewise, the odds of having symptoms at birth were higher when mothers were HLA-G del/del, although not significant (not shown). Next, given the role of HLA-G during CMV infection, the HLA-G del/del polymorphism was assessed in relation to CMV viral load. For this purpose, the study group was divided into two groups according to the viral load measured in DBS namely low (< 500 IU/ml) and high (> 500 IU/ml) viral load groups. A higher percentage of mothers with HLA-G del/del was observed in the high viral load group compared to the low viral load group (41.4 and 12.0%, respectively, *p* = 0.008), whereas this was not observed in the children (30.0 and 32.0% respectively, *p* = 0.852) (Table S3 in Supplementary Material).

**Table 3:** HLA-G and congenital CMV infection (cCMV) clinical outcome.

	Symptoms at birth			Long-term impairments (LTI)		
	Symptomatic % (*n* = 19)	Asymptomatic % (*n* = 77)	*p*-Value (Chi)	LTI (≥1)^a^ % (*n* = 27)	No LTI^b^ % (*n* = 69)	*p*-Value (Chi)
**HLA-G genotype**						
*Mother*			0.174			0.023*
del/del	52.6	29.9		55.6	26.1	
ins/ins	10.5	15.6		11.1	15.9	
del/ins	36.8	54.5		33.3	58	
*Child*			0.459			0.514
del/del	26.3	32.5		37	29	
ins/ins	10.5	19.5		11.1	20.3	
del/ins	63.2	48.1		51.9	50.7	

HLA-G del mm^c^	5.3	10.4	0.683^j^	3.7	11.6	0.437^j^
HLA-G ins mm^d^	31.6	11.7	0.070^j^	25.9	11.6	0.116^j^

HLA-G del ms^e^	5.3	14.3	0.449^j^	3.7	15.9	0.169^j^
HLA-G ins ms^f^	5.3	14.3	0.449^j^	7.4	14.5	0.500^j^

*^a^Any long-term impairment, in one or more domains of impairments: hearing, visual, neurologic, motor, cognitive, and speech-language*.

*^b^Absence of any long-term impairment*.

*^c^HLA-G del mm: HLA-G deletion mismatches (mm), the mother is homozygous for HLA-G insertion and the child is heterozygous*.

*^d^HLA-G ins mm: HLA-G insertion mm, the mother is homozygous for HLA-G deletion and the child is heterozygous*.

*^e^HLA-G del ms: HLA-G deletion missing-self, the mother is heterozygous and the child is homozygous for HLA-G insertion*.

*^f^HLA-G ins ms: HLA-G insertion missing-self, the mother is heterozygous and the child is homozygous for HLA-G deletion*.

*^j^Fischer’s exact test used*.

*p < 0.05.

Finally, we evaluated whether the child’s genotype plays a role in cCMV outcome. The genotype frequencies of the HLA-C groups, and HLA-G alleles in the children were in HWE in all groups, whereas the HLA-E alleles were not, both in the symptomatic and asymptomatic groups (*p* = 0.037 and *p* = 0.024, respectively) (Table S2 in Supplementary Material). A higher percentage of children heterozygous for HLA-E was observed in the symptomatic group compared to the asymptomatic group whereas the percentage of homozygotes, either HLA-E*0101 or HLA-E*0103, was lower (Table 4). HLA-E*0103 is associated with higher protein levels (40); therefore, our findings suggest that the functional difference of the two alleles is not associated with clinical outcome.

**Table 4:** HLA-E and congenital CMV infection (cCMV) clinical outcome.

	Symptoms at birth			Long-term impairments (LTI)		
	Symptomatic % (*n* = 19)	Asymptomatic % (*n* = 76)	*p*-Value (Chi)	LTI (≥1)^a^ % (*n* = 26)	No LTI^b^ % (*n* = 69)	*p*-Value (Chi)
HLA-E genotype						
*Mother*			0.499			0.809
0101/0101	21.1	27.6		23.1	27.5	
0103/0103	36.8	23.7		30.8	24.6	
0101/0103	42.1	48.7		46.2	47.8	
*Child*^g^			0.015*			0.714
0101/0101	15.8	35.5		33.3	30.9	
0103/0103	10.5	27.6		18.5	26.5	
0101/0103	73.7	36.8		48.1	42.6	

HLA-E*0101 mm^c^	36.8	11.8	**0.016**^h*^	26.9	13	0.129^h^
HLA-E*0103 mm^d^	15.8	9.2	0.413^h^	15.4	8.7	0.453^h^

HLA-E*0101 ms^e^	10.5	17.1	0.728^h^	15.4	15.9	1.000^h^
HLA-E*0103 ms^f^	10.5	17.1	0.728^h^	23.1	13	0.343^h^

*^a^Any long-term impairment, in one or more domains of impairments: hearing, visual, neurologic, motor, cognitive, and speech-language*.

*^b^Absence of any long-term impairments*.

^c^HLA-E*0101 mm: HLA-E*0101 mismatches (mm), the mother is homozygous for HLA-E*0103 and the child is heterozygous.

^d^HLA-E*0103 mm: HLA-E*0103 mm, the mother homozygous for HLA-E*0101 and the child is heterozygous.

^e^HLA-E*0101 ms: HLA-E*0101 missing-self, the mother is heterozygous and the child is homozygous for HLA-E*0103.

^f^HLA-E*0103 ms: HLA-E*0103 missing-self, the mother is heterozygous and the child is homozygous for HLA-E*0101.

*^g^N = 27 neonates with LTI and N = 68 neonates without LTI*.

*^h^Fischer’s exact test used*.

* p < 0.05.

### Maternal–Fetal HLA-C, HLA-E, and HLA-G mm in Relation to cCMV Clinical Outcome

We next investigated whether maternal–fetal HLA mm are associated with a worse cCMV outcome. For this purpose, mm were calculated on the basis of the presence of an Ag in the fetus, which was absent in the mother because the inherited paternal antigen (IPA) was different from both maternal alleles (Table 5). The mm were compared between children symptomatic and asymptomatic at birth, as well as between children who developed LTI and those who did not. A significantly higher percentage of HLA-C2 mm was observed in the symptomatic group compared to the asymptomatic group (Table 2). Most likely, the mm derived from the higher percentage of mothers homozygous for HLA-C1 while a higher percentage of symptomatic neonates was heterozygous (Table 2). Therefore, the functional consequence of HLA-C2 absence in the mother might be the main risk factor, even though the odds of having symptoms at birth were significantly higher as well with HLA-C2 mm (OR = 3.923, 95% CI 1.166–13.201, *p* = 0.027).

**Table 5:** Definitions: mismatches (mm), missing-self (ms), and non-inherited maternal antigens (NIMAs).

Genotype combinations^a^		Maternal perspective		Child perspective
		
Maternal genotype	Fetal genotype	Mismatch (mm)^b^	Missing-self (ms)^c^	NIMAs^d^
a/b	a/a	No	Yes	Yes
a/b	b/b	No	Yes	Yes
a/b	a/c	Yes	Yes	Yes
a/b	c/b	Yes	Yes	Yes
a/a	a/b	Yes	No	No
b/b	a/b	Yes	No	No
a/a	a/a	No	No	No
a/b	a/b	No	No	No
b/b	b/b	No	No	No

*^a^Combinations of maternal and child HLA genotype by using 3 hypothetical genes (a, b, c)*.

*^b^mm are defined as the antigen (Ag) that the child has but the mother does not have, because the inherited paternal antigen (IPA) is different from both maternal alleles*.

*^c^Missing-self (ms) is defined as the Ag that the mother has and the child does not because the inherited paternal antigen (IPA) differs from the non-inherited maternal antigen (NIMAs)*.

*^d^NIMAs are defined as the Ag that the child did not inherit but is exposed to, due to maternal microchimerism*.

To exclude the possibility that symptoms at birth were associated with specific HLA-C KIR combinations rather than maternal genotypes or maternal–fetal mm, the number of HLA-C1/HLA-C2 KIR epitope combinations were analyzed. No differences in preferential fetal HLA-C/maternal KIR combinations were observed (Table 2). In addition, a significantly higher percentage of HLA-E*0101 mm was observed in the symptomatic group compared to the asymptomatic (Table 4). The HLA-E mm may be driven by the neonatal aberrant distribution of the genotypes. However, because the difference in HLA-E genotypes would probably have no functional consequences, the mm may be the primary risk factor. Indeed, the odds of developing symptoms at birth were significantly higher when HLA-E*0101 mm were present (OR = 4.343, 95% CI 1.357–13.897, *p* = 0.013). In addition, the HLA-E*0101 mm was assessed in relation to CMV viral load, and a higher percentage of HLA-E*0101 mm was found in the high viral load group compared to the low viral load group (23.2% and 0.0% respectively, *p* = 0.005) (Table S3 in Supplementary Material). Finally, no differences in HLA-G mm were observed with respect to symptoms nor to LTI development (Table 3).

### HLA-C, HLA-E, and HLA-G Missing-Self/NIMAs in Relation to cCMV Clinical Outcome

We finally assessed whether missing-self/NIMAs influence cCMV clinical outcome. For this purpose, missing-self was defined as an Ag in the mother, which was absent in the child because the IPA was different from the NIMA (Table 5). From the mothers’ perspective, missing-self implies a mechanism of recognition by dNK of trophoblasts lacking maternal self-molecules. Missing-self does not necessarily indicate mm because the IPA could still be the same as the inherited maternal Ag (Table 5). The maternal Ag, which was missing in the child, is considered by the child a NIMA. Therefore, despite being the same aforementioned Ag, other mechanisms than recognition by dNK of missing-self are in place. Hence, we will refer to this Ag, triggering different responses, as missing-self/NIMAs. In our cohort, there were no significant differences in HLA-C, HLA-E, and HLA-G missing-self/NIMAs in relation to symptoms at birth. However, for LTI development, a significantly higher percentage of HLA-C missing-self/NIMAs, but not of HLA-E and HLA-G missing-self/NIMAs, was observed (Tables 2–4). The aforementioned results suggest that the HLA-C missing-self/NIMA may be considered as a risk factor for LTI development, rather than for symptoms at birth. Indeed, the odds of developing LTI was significantly higher when the child had HLA-C mismatched NIMAs (OR 13.3, 95% CI 1.701–104.535, *p* = 0.014).

## Discussion

To gain more insights into cCMV pathogenesis and its clinical consequences, the role of HLA-C, HLA-E, and HLA-G genotypes was evaluated in a large cohort of children with cCMV and their mothers. HLA-C, HLA-E, and HLA-G are the only HLA molecules expressed by the trophoblasts and, therefore, they might play a role in cCMV outcome. To systematically discuss the findings, they will be described in relation to the compartments involved in the virus–host interaction, which are maternal, placental, fetal, and child.

**Figure 1:**
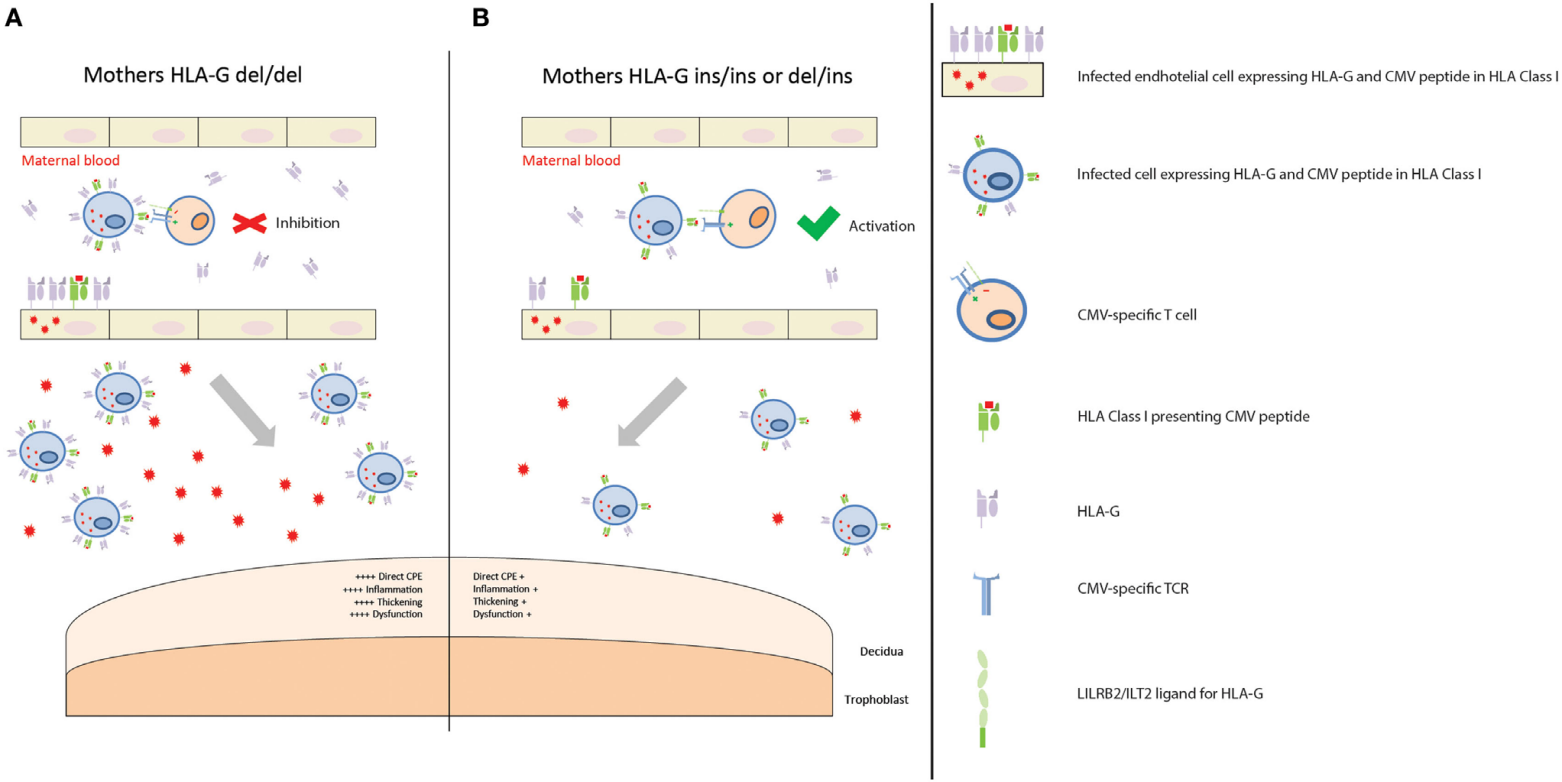
Placental CMV infection according to maternal HLA-G genotype. **(A)** Mothers homozygous for HLA-G deletion are predisposed to having higher levels of soluble HLA-G (sHLA-G), and possibly membrane-bound HLA-G, proteins in those tissues where HLA-G is expressed, such as endothelial cells and monocytes/macrophages. When maternal T cells encounter an infected cell with higher levels of HLA-G, it is inhibited. This creates a favorable environment for CMV replication and dissemination. Consequently, more extensive infection of the placenta leads to increased CPE, inflammation, thickening, and dysfunction. This, in turn, contributes to a worse outcome. **(B)** Mothers homozygous for HLA-G insertion, or heterozygous, are predisposed to having lower levels of sHLA-G, and possibly membrane-bound HLA-G, proteins. When maternal T cells encounter an infected cell with lower levels of HLA-G protein, it is activated. CMV replication and dissemination is, therefore, contained. Consequently, lower numbers of infected cells and viral loads reach the placenta, with a reduced CPE, inflammation, thickening, and dysfunction. This in turn contributes to a better outcome.

First of all, the nature of maternal infection and her immune response can influence cCMV outcome. In our cohort, the maternal HLA-G del/del genotype was associated with a worse cCMV outcome. This could be explained by the immunosuppressive effect of this genotype which is associated with the presence of higher HLA-G protein levels, soluble and possibly membrane-bound (52, 53). Indeed, HLA-G can inhibit various immune cell subsets (16, 17). The hypothetical model to account for maternal HLA-G genotype in relation to cCMV outcome is depicted in Figure 1. In addition, CMV induces upregulation of HLA-G in order to escape the host defense and, after an initial local replication, CMV dissemination is likely to be cell-associated, occurring mainly through endothelial and monocytes/macrophages (54). Hence, higher HLA-G protein levels might create a favorable environment for CMV, facilitating replication and dissemination. Interestingly, this HLA-G del/del polymorphism has been associated with active CMV infection with higher viral loads in children (29). In our cohort, maternal samples during pregnancy were not available. However, neonatal CMV viral load determined in DBS was related to the HLA-G genotype of the mother, as reflected by the higher percentage of mothers HLA-G del/del in the high viral load group compared to the low viral load group (Table S3 in Supplementary Material). This suggests that a reduced maternal control of CMV infection may increase the viral loads at the placenta and, consequently, in the fetus. Placental CMV infection triggers an inflammatory response that alters the trophoblast, inducing placental dysfunction and fetal impairments, such as intrauterine growth retardation (55–57). Interestingly, in a group of pregnant women with primary CMV infection and neonates with CMV disease, the placental thickness was increased (58). In addition, in another cohort of pregnant women with primary CMV infection, increased sHLA-G levels were observed in both maternal serum and amniotic fluid of symptomatic fetuses (30). However, no information was available on the maternal and child HLA-G genotypes.

**Figure 2:**
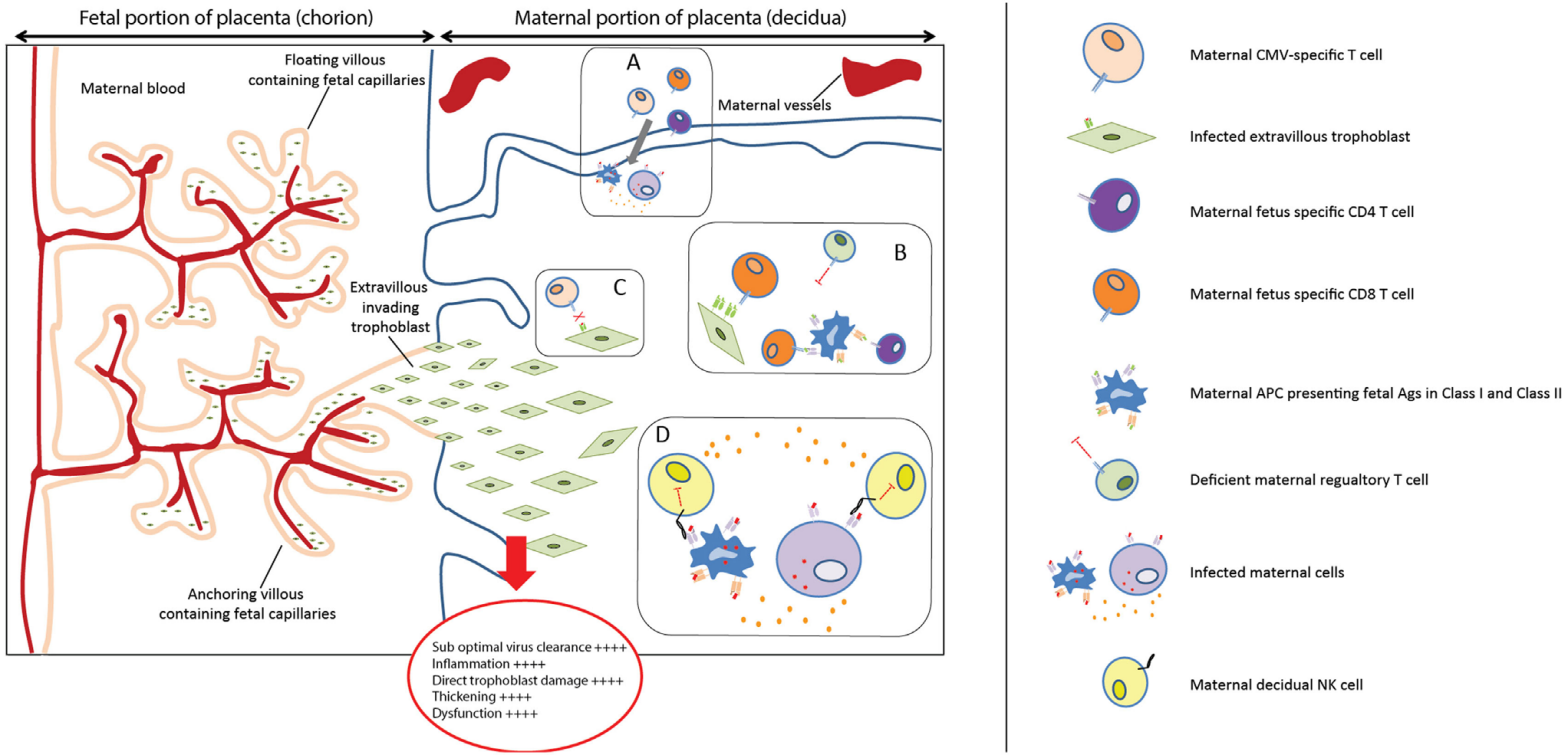
Placental immunopathology. **(A)** Viral infections can increase the levels of pro-inflammatory cytokines and chemokines and increase the influx of T cells in decidual tissues (60, 61). **(B)** The regulatory mechanisms might not be able to efficiently modulate the maternal fetus-specific lymphocytes, specific for HLA-C and HLA-E, which can, therefore, directly or indirectly recognize trophoblast cells. **(C)** HLA-C and HLA-E mismatches (mm) could prevent an optimal viral clearance because maternal CMV-specific cells would not efficiently recognize CMV presented in the context of allo-HLA. **(D)** In the absence of maternal HLA-C2, the balance between activating and inhibitory signals on dNK cells may be more skewed toward dNK cells activation, because HLA-C1 has less inhibitory capacity on dNK. This activation could in turn promote a pro-inflammatory response, exacerbating placental immunopathology.

Vertical transmission occurs through placental infection, which therefore was considered after the maternal immune response. The hypothetical model to account for our findings in the placenta in relation to cCMV outcome is shown in Figure 2. First of all, a significantly higher percentage of symptomatic neonates had HLA-E*0101 mm, and a higher extent of HLA-C mm. HLA mm have been shown to result in the occurrence of maternal fetus-specific T cells, both in the maternal peripheral blood and at the maternal–fetal interface (20, 59). In normal conditions, this does not impair pregnancy because there is a parallel increase of regulatory mechanisms modulating such responses (20). However, viral infections can increase the levels of pro-inflammatory cytokines, chemokines, and the influx of T cells in decidual tissues (60, 61). In this situation, the regulatory mechanisms might not be able to efficiently inhibit allogeneic lymphocytes (19), which could damage the placenta. Furthermore, because CMV peptides can be presented in the context of HLA-C and HLA-E (62–64), mm could lead to suboptimal viral clearance at the placenta, as maternal CMV-specific cells would not efficiently recognize CMV presented in the context of allo-HLA. This has been described in allogeneic hematopoietic stem cell transplantation, where the clinical activity of donor-derived virus-specific T cells can be abolished if the immunodominant T cells are restricted by HLA not shared by the host (65). Consequently, this may result in higher placental and fetal viral loads. Indeed, a higher percentage of maternal–fetal HLA-E*0101 mm was found in the high viral load group compared to the low viral load group, and the same was observed for HLA-C mm (76.8 and 56.0% respectively, *p* = 0.049) (Table S3 in Supplementary Material). Finally, dNK cells are the predominant leukocyte population at the placenta, and they play a central role in the immune cross-talk and in the placentation process. The combination of maternal AA KIR with fetal HLA-C2 was associated with increased risk of pre-eclampsia, as it led to the absence of activated dNK and poor placentation (23). Indeed, HLA-C2 has a stronger inhibition capacity when binding to its inhibitory KIR (KIR2DL1) than C1 with its corresponding receptor (KIR2DL2/3) (23, 50, 51). In our cohort, clinical outcome was not associated with specific maternal KIR-fetal HLA-C combinations. Rather, the absence of maternal HLA-C belonging to the C2 group was associated with a worse cCMV outcome at birth. In the absence of HLA-C2, the activating signals on dNK cells may prevail the inhibitory signals, which in turn promote a pro-inflammatory response. In addition, it has been shown that expression of KIR2DS1 by dNK increases their cytotoxic function toward infected maternal decidual stromal cells, which could possibly lead to a reduction of placental virus-induced pathology (24). In our cohort, the presence of the maternal gene KIR2DS1 was not associated with a better short- and long-term outcome (Table S4 in Supplementary Material). However, these results would need to be confirmed as the lack of statistical power may have been a limiting factor in detecting a small effect. Taken together, the aforementioned mechanisms may contribute to the complex multifactorial process of placental immunopathology and dysfunction, which has a direct impact on outcome at birth. Importantly, besides the fact that placental inflammation has been described in relation to cCMV (55–57), prematurity has also been associated with chronic placental inflammation in the absence of infections (66). Given the relatively high percentage of premature infants with cCMV in our cohort, we evaluated whether the aforementioned markers shown to be associated with symptoms may have been influenced by this. After excluding the 11 premature infants, a slight change in *p*-values, but with the same trend of percentages, was observed in relation to symptoms at birth (data not shown), most likely due to the lack of statistical power. This suggests that we cannot fully exclude that the relatively high percentage of premature infants in the symptomatic group partially influenced the association between the aforementioned biomarkers and symptoms. Taking into account the association between cCMV and prematurity (67), it may be plausible to assume that prematurity is an effect of both cCMV and the aforementioned HLAs.

**Figure 3:**
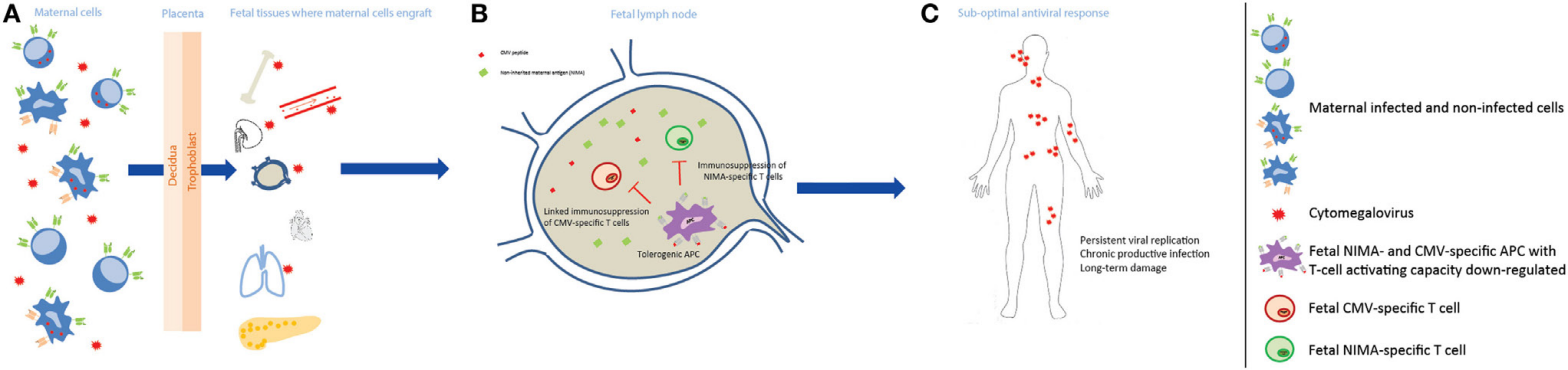
Non-inherited maternal antigen (NIMA) effect and linked immunosuppression in congenital CMV infection long-term outcome. **(A)** During pregnancy, maternal cells pass through the placenta to the fetus and can engraft in several fetal tissues, which may be infected by CMV, and persist at least till adulthood. Maternal cells can be either CMV infected or not, and can carry HLA Ags that the child does not have, i.e., NIMAs. **(B)** After NIMAs recognition by fetal T cells, tolerogenic APCs, with reduced T cell-activating capacity, are induced in order to suppress a fetal antimaternal immune response. These tolerogenic APCs may be also specific for CMV, as maternal cells can be infected or can reside in infected fetal tissues, and suppress CMV-specific T cells (linked immunosuppression). **(C)** The antiviral response in the child is therefore less efficient, possibly leading to a more persistent viral replication with a chronic productive infection, and tissue damage.

The child’s immune response was considered after the placental compartment, and the hypothetical model to account for our findings in relation to cCMV outcome is shown in Figure 3. Although certain cCMV clinical consequences are present directly after birth, some of the permanent impairments have an onset in the first years after birth, or may progress during childhood (1). The late-onset hearing loss is commonly considered as the result of a chronic disease syndrome, of which the molecular mechanisms have not been elucidated yet, though data suggest that they are due to a chronic productive infection throughout childhood (7, 8). For this reason, the child’s immune response to CMV may play a central role in preventing LTI. In our cohort, a high percentage of children with LTI had maternal–fetal HLA-C NIMAs. Their role in LTI development may lie in the tolerance induced in the fetus toward NIMAs. Indeed, during pregnancy and after birth, the child is exposed to maternal allogeneic cells because of the transfer of maternal cells to the fetus, namely maternal microchimerism (68, 69). The developing neonatal immune system does not consider NIMAs as non-self, but rather develops long-lasting regulatory mechanisms to prevent an immune response against NIMAs. The NIMA effect has mainly been shown in transplantation. The survival rate of kidney grafts with a mismatched Ag identical to the recipient’s NIMA was better than in situations where the mismatched Ag was not a NIMA (70, 71). Similar effects have been shown in case of stem cell, cord blood, and bone marrow transplantation (72–74). Fetal T cells can recognize NIMAs presented by maternal cells *via* the shared HLA, or by fetal APC in the context of HLA class I and class II. One of the first steps in the tolerance toward NIMAs is the induction of NIMA-specific Tregs, which suppress the fetal antimaternal immunoresponse and persist at least till early adulthood (75, 76). In addition, tolerogenic APCs presenting NIMAs are induced, and suppress NIMAs-specific T cells (77, 78). These mechanisms may influence the child’s immune response. First, the NIMA-specific Tregs may create a general tolerogenic environment that indirectly impair the immune response to CMV. Second, through a mechanism called linked immunosuppression, a certain level of tolerance specific for CMV can be induced (78). Indeed, fetal APCs could present both NIMAs and CMV Ag, as maternal cells can be infected or because maternal cells reside in infected fetal tissues. This may result in a less efficient antiviral response during childhood, which possibly leads to a more persistent viral replication and tissue damage. Therefore, while the NIMA effect may be beneficial in transplantation, it could prevent an optimal antiviral response due to a partial tolerance to CMV as well. Similar to symptoms at birth, we evaluated whether the relatively high percentage of premature infants in the symptomatic group may have influenced the results relative to LTI. After excluding the premature infants, the results and significance did not change (data not shown), suggesting that the relatively high percentage of premature does not influence the association between HLA-C NIMAs and LTI development.

This study is not without limitations. First of all, potential effects of CMV on the expression of the studied HLA Ags could not be taken into account. Indeed, CMV has developed strategies to evade host immunity and to establish latency, e.g., by down-regulating classical HLA molecules and upregulating non-classical HLA (54, 79–81). Second, a CMV-independent role of these HLA combinations in adverse pregnancy outcome cannot be totally excluded. However, we did not observe the previously described HLA KIR combinations associated with pregnancy complications, further suggesting that our observations specifically apply to cCMV.

To the best of our knowledge, this is the first study on maternal–fetal HLA in a large cohort of children with cCMV. If our hypotheses are correct, symptoms at birth are mainly caused by the immunopathology that takes place at the maternal–fetal interface, as a result of a multifactorial process of suboptimal viral clearance, enhanced allo-reactivity, and increased inflammation. Whereas the inefficient long-term control of CMV infection, which plays a role in LTI development, might have been partially caused by the NIMA effect. This study gives useful insights and generates new hypotheses on cCMV pathogenesis in all compartments involved during cCMV. Furthermore, if confirmed in other cohorts, these findings could be evaluated as potential prognostic markers for clinical outcome. Indeed, a reliable marker for cCMV outcome could provide the means to introduce the long-debated newborn screening program for CMV in DBS by defining subgroups of neonates that would benefit from clinical, audiological follow-up, and possibly antiviral treatment (82).

## Ethics Statement

This study was approved by the Medical Ethics Committee of the Leiden University Medical Center, and all the parents of the children included have given written informed consent in accordance with the Declaration of Helsinki.

## Author Contributions

RR and AV designed research; RR, DR, and ME performed and supervised the experiments; RR and GH analyzed the data; RR and FC interpreted the results in relation to the possible mechanisms; RR and AV wrote the manuscript; FC, GH, AK, and ME revised the manuscript critically.

## Conflict of Interest Statement

The authors declare that the research was conducted in the absence of any commercial or financial relationships that could be construed as a potential conflict of interest.

## Abbreviations

cCMV, congenital CMV infection; LTI, long-term impairments; dNK, decidual natural killer cells; DBS, dried blood spots; mm, mismatches; NIMAs, non-inherited maternal antigens; IPA, inherited paternal antigen; Ags, Antigens; HLA-G deletion, HLA-G del/del; sHLA-G, soluble HLA-G.
