# Supplementary material for: Congenital Cytomegalovirus Infection: Maternal–Child HLA-C, HLA-E, and HLA-G Affect Clinical Outcome
Source: Front Immunol. 2018 Jan 5;8:1904. doi: 10.3389/fimmu.2017.01904 (PMC5760553; doi:10.3389/fimmu.2017.01904)
Supplement: Supplementary file 1 [file table_1.docx]

Supplementary Material

Congenital Cytomegalovirus Infection: Maternal-Child HLA-C, HLA-E and HLA-G Affect Clinical Outcome

Roberta Rovito^*^, Frans H.J. Claas , Geert W. Haasnoot , Dave L. Roelen, Aloys C.M. Kroes, Michael Eikmans, Ann C.T.M Vossen

*** Correspondence:**Roberta Rovito
[R.Rovito@lumc.nl](mailto:R.Rovito@lumc.nl)

# Supplementary Tables

**TABLE S1** HLA-C frequency in 5604 randomly selected healthy Dutch blood donors

| **HLA-C alleles** | **Positive** | **Negative** | **%** |
| --- | --- | --- | --- |
| C*01 | 323 | 5281 | 5,8% |
| C*02 | 575 | 5029 | 10,3% |
| C*03 | 1665 | 3939 | 29,7% |
| C*04 | 1287 | 4317 | 23,0% |
| C*05 | 764 | 4840 | 13,6% |
| C*06 | 898 | 4706 | 16,0% |
| C*07 | 3140 | 2464 | 56,0% |
| C*08 | 233 | 5371 | 4,2% |
| C*12 | 466 | 5138 | 8,3% |
| C*14 | 117 | 5487 | 2,1% |
| C*15 | 287 | 5317 | 5,1% |
| C*16 | 343 | 5261 | 6,1% |
| C*17 | 97 | 5507 | 1,7% |
| C*18 | 2 | 5602 | 0,0% |
