# Supplementary material for: Congenital Cytomegalovirus Infection: Maternal–Child HLA-C, HLA-E, and HLA-G Affect Clinical Outcome
Source: Front Immunol. 2018 Jan 5;8:1904. doi: 10.3389/fimmu.2017.01904 (PMC5760553; doi:10.3389/fimmu.2017.01904)
Supplement: Supplementary file 2 [file table_2.docx]

Supplementary Material

Congenital Cytomegalovirus Infection: Maternal-Child HLA-C, HLA-E and HLA-G Affect Clinical Outcome

Roberta Rovito^*^, Frans H.J. Claas , Geert W. Haasnoot , Dave L. Roelen, Aloys C.M. Kroes, Michael Eikmans, Ann C.T.M Vossen

*** Correspondence:**Roberta Rovito
[R.Rovito@lumc.nl](mailto:R.Rovito@lumc.nl)

# Supplementary Tables

**TABLE S2** Hardy-Weinberg Equilibrium and cCMV clinical outcome

|  | **Symptoms at birth** | | | | | | **Long-term impairments (LTI)** | | | | | |
| --- | --- | --- | --- | --- | --- | --- | --- | --- | --- | --- | --- | --- |
|  | **Sympt.** | | | **Asympt.** | |  | **LTI ( ≥ 1)** | |  | **No LTI** | |  |
|  | Obs.^1^ | Exp.^2^ | p-value^3^ | Obs. | Exp. | p-value | Obs. | Exp. | p-value | Obs. | Exp. | p-value |
| **HLA-C Genotype** |  |  |  |  |  |  |  |  |  |  |  |  |
|  |  |  |  |  |  |  |  |  |  |  |  |  |
| *Mother* |  |  | **0.046** |  |  | 0.535 |  |  | 0.239 |  |  | 0.581 |
| C1C2 | 4 | 7.4 |  | 40 | 37.3 |  | 10 | 13 |  | 34 | 31.9 |  |
| C1C1 | 12 | 10.3 |  | 23 | 24.3 |  | 8 | 6.5 |  | 27 | 28.1 |  |
| C2C2 | 3 | 1.3 |  | 13 | 14.3 |  | 8 | 6.5 |  | 8 | 9.1 |  |
|  |  |  |  |  |  |  |  |  |  |  |  |  |
| *Child* |  |  | 0.342 |  |  | 0.655 |  |  | 0.945 |  |  | 0.917 |
| C1C2 | 10 | 8.2 |  | 35 | 36.9 |  | 13 | 12.8 |  | 32 | 32.4 |  |
| C1C1 | 8 | 8.9 |  | 27 | 26.1 |  | 8 | 8.1 |  | 27 | 26.8 |  |
| C2C2 | 1 | 1.9 |  | 14 | 13.1 |  | 5 | 5.1 |  | 10 | 9.8 |  |
|  |  |  |  |  |  |  |  |  |  |  |  |  |
| **HLA-E Genotype** |  |  |  |  |  |  |  |  |  |  |  |  |
|  |  |  |  |  |  |  |  |  |  |  |  |  |
| *Mother* |  |  | 0.552 |  |  | 0.828 |  |  | 0.716 |  |  | 0.723 |
| 0101/0101 | 4 | 3.4 |  | 21 | 20.5 |  | 6 | 5.5 |  | 19 | 18.3 |  |
| 0103/0103 | 7 | 6.4 |  | 18 | 17.5 |  | 8 | 7.5 |  | 17 | 16.3 |  |
| 0101/0103 | 8 | 9.3 |  | 37 | 37.9 |  | 12 | 12.9 |  | 33 | 34.5 |  |
|  |  |  |  |  |  |  |  |  |  |  |  |  |
| *Child* |  |  | **0.037** |  |  | **0.024** |  |  | 0.936 |  |  | 0.230 |
| 0101/0101 | 3 | 5.3 |  | 27 | 22.1 |  | 9 | 8.9 |  | 21 | 18.5 |  |
| 0103/0103 | 2 | 4.3 |  | 21 | 16.1 |  | 5 | 4.9 |  | 18 | 15.5 |  |
| 0101/0103 | 14 | 9.5 |  | 28 | 37.8 |  | 13 | 13.2 |  | 29 | 33.9 |  |
| **HLA-G Genotype** |  |  |  |  |  |  |  |  |  |  |  |  |
|  |  |  |  |  |  |  |  |  |  |  |  |  |
| *Mother* |  |  | 0.649 |  |  | 0.318 |  |  | 0.379 |  |  | 0.154 |
| del/del | 10 | 9.6 |  | 23 | 25.1 |  | 15 | 14.1 |  | 18 | 20.9 |  |
| ins/ins | 2 | 1.6 |  | 12 | 14.1 |  | 3 | 2.1 |  | 11 | 13.9 |  |
| del/ins | 7 | 7.8 |  | 42 | 37.7 |  | 9 | 10.8 |  | 40 | 34.1 |  |
|  |  |  |  |  |  |  |  |  |  |  |  |  |
| *Child* |  |  | 0.198 |  |  | 0.844 |  |  | 0.561 |  |  | 0.853 |
| del/del | 5 | 6.4 |  | 25 | 24.6 |  | 10 | 10.7 |  | 20 | 20.4 |  |
| ins/ins | 2 | 3.4 |  | 15 | 14.6 |  | 3 | 3.7 |  | 14 | 14.4 |  |
| del/ins | 12 | 9.3 |  | 37 | 37.8 |  | 14 | 12.6 |  | 35 | 34.2 |  |

^1^ Obs: observed number of subjects; ^2^ Exp: expected number of subjects; ^3^ Chi-square test.
