# Supplementary material for: Congenital Cytomegalovirus Infection: Maternal–Child HLA-C, HLA-E, and HLA-G Affect Clinical Outcome
Source: Front Immunol. 2018 Jan 5;8:1904. doi: 10.3389/fimmu.2017.01904 (PMC5760553; doi:10.3389/fimmu.2017.01904)
Supplement: Supplementary file 3 [file table_3.docx]

Supplementary Material

Congenital Cytomegalovirus Infection: Maternal-Child HLA-C, HLA-E and HLA-G Affect Clinical Outcome

Roberta Rovito^*^, Frans H.J. Claas , Geert W. Haasnoot , Dave L. Roelen, Aloys C.M. Kroes, Michael Eikmans, Ann C.T.M Vossen

*** Correspondence:**Roberta Rovito
[R.Rovito@lumc.nl](mailto:R.Rovito@lumc.nl)

# Supplementary Tables

**TABLE S3** HLA-G deletion genotype, HLA-E and HLA-C mm in relation to viral load

|  | **Viral load^1^** | | |
| --- | --- | --- | --- |
|  | Low^2^  % | High^3^  % | p-value  Chi |
|  | n = 25 | n = 70 |  |
| **HLA-G del/del mother^4^** | 12.0 | 41.4 | **0.008*** |
| **HLA-G del/del child^5^** | 32.0 | 30.0 | 0.852 |
| **HLA-E*0101 mm^6^** | 0.00 | 23.2 | **0.005**˜* |
| **HLA-C mm^7^** | 56.0 | 76.8 | **0.049*** |

^1^ The study group was divided in two groups according to the CMV viral load measured in DBS. For one child the DBS was not available and the viral load could not be assessed; ^2^ Low: viral loads in DBS < 500 IU/ml; ^3^ High: viral loads in DBS ≥ 500 IU/ml; ^4^ Mothers homozygous for HLA-G deletion; ^5^ Children homozygous for HLA-G deletion; ^6^ HLA-E*0101 mm: HLA-E*0101 mismatches, the mother is homozygous for HLA-E*0103 and the child is heterozygous; ^7^ HLA-C mm: HLA-C mismatches; ^6,7^ N high group = 69. ˜ Fischer’s exact test used. * p < 0.05.
