# Supplementary material for: Congenital Cytomegalovirus Infection: Maternal–Child HLA-C, HLA-E, and HLA-G Affect Clinical Outcome
Source: Front Immunol. 2018 Jan 5;8:1904. doi: 10.3389/fimmu.2017.01904 (PMC5760553; doi:10.3389/fimmu.2017.01904)
Supplement: Supplementary file 4 [file table_4.docx]

Supplementary Material

Congenital Cytomegalovirus Infection: Maternal-Child HLA-C, HLA-E and HLA-G Affect Clinical Outcome

Roberta Rovito^*^, Frans H.J. Claas , Geert W. Haasnoot , Dave L. Roelen, Aloys C.M. Kroes, Michael Eikmans, Ann C.T.M Vossen

*** Correspondence:**Roberta Rovito
[R.Rovito@lumc.nl](mailto:R.Rovito@lumc.nl)

# Supplementary Tables

**TABLE S4** Individual KIRs and cCMV clinical outcome

|  | **Symptoms at birth** | | | **Long-term impairments (LTI)** | | |
| --- | --- | --- | --- | --- | --- | --- |
| **KIRs** | Symptomatic  %  n = 19 | Asymptomatic  %  n = 73 | p-value  Chi | LTI ( ≥ 1)^1^  %  n = 25 | No LTI^2^  %  n = 67 | p-value  Chi |
| KIR2DL1 | 100.0 | 89.0 | 0.198^~^ | 100.0 | 88.1 | 0.102^~^ |
| KIR2DL2 | 47.4 | 53.4 | 0.638 | 44.0 | 55.2 | 0.338 |
| KIR2DL3 | 100.0 | 86.3 | 0.115^~^ | 92.0 | 88.1 | 0.723^~^ |
| KIR2DL5 | 42.1 | 47.9 | 0.649 | 52.0 | 44.8 | 0.537 |
| KIR2DS1 | 42.1 | 34.2 | 0.525 | 40.0 | 34.3 | 0.614 |
| KIR2DS2 | 47.4 | 54.8 | 0.563 | 44.0 | 56.7 | 0.277 |
| KIR2DS3 | 21.1 | 21.9 | 1.000^~^ | 32.0 | 17.9 | 0.145 |
| KIR2DS4 | 100.0 | 97.3 | 1.000^~^ | 100.0 | 97.0 | 1.000^~^ |
| KIR2DS5 | 36.8 | 30.1 | 0.575 | 40.0 | 28.4 | 0.285 |
| KIR3DL1 | 100.0 | 97.3 | 1.000^~^ | 100.0 | 97.0 | 1.000^~^ |
| KIR3DS1 | 42.1 | 35.6 | 0.602 | 44.0 | 34.3 | 0.393 |

^1^ Any long-term impairment, in one or more domains of impairments: hearing, visual neurologic, motor, cognitive, and speech-language; ^2^ Absence of any long-term impairment; ˜ Fischer’s exact test used.
